# A case of severe sciatica caused by a lymphocele after renal transplantation

**DOI:** 10.1186/s40981-022-00527-2

**Published:** 2022-05-30

**Authors:** Naoto Yamada, Motoi Kumagai, Kenji S Suzuki

**Affiliations:** Department of Anesthesiology, School of Medicine, Iwate Medical University, 2-1-1 Idai-dori, Yahaba-cho, Shiwa-gun, Iwate, 028-3695 Japan

**Keywords:** Sciatica, Renal transplantation, Lymphocele, Piriformis muscle syndrome

## Abstract

**Background:**

Sciatica is commonly caused by lumbar spinal disease. However, it can also be caused by tumors, infectious diseases, or muscle entrapment. We present a case of sciatica caused by a lymphocele after renal transplantation.

**Presentation:**

A 50-year-old man who had undergone renal transplantation presented with sciatica and low back pain without leg edema. The patient was diagnosed with lumbar disc herniation during the first medical examination. Regardless of the treatment, the symptoms were exacerbated and red flag signs of low back pain were observed. Compression of the sciatic nerve by the lymphocele was confirmed by computed tomography. The sciatica was improved by ethanol injection for the lymphocele.

**Conclusions:**

We encountered a rare case of severe sciatica without edema caused by lymphocele after renal transplantation. Careful examination is required to make a different diagnosis of lymphocele from other lumbar spinal diseases.

## Background

Sciatica is caused by sciatic nerve compression, resulting in severe leg pain and functional limitations. These symptoms are often derived from lumbar spinal disease such as lumbar disc herniation, and the rate of occurrence is estimated to be 13-40% over a lifetime [1]. In clinical practice, pain clinicians and orthopedists commonly treat sciatica as lumbar spinal disease. However, rare causes such as neoplastic or infectious disease and muscle entrapment can present with symptoms similar to those of lumbar spinal disease [1–3]. Symptomatic lymphocele typically presents with unilateral leg edema due to iliac vein compression [4], and sciatica without leg edema caused by lymphocele after renal transplantation is atypical and may easily be confused with those of lumbar spinal diseases. We present a case of sciatica caused by a lymphocele after renal transplantation.

## Case presentation

A 50-year-old man with diabetic nephropathy had undergone renal transplantation five months prior to consultation. He was receiving immunosuppressants, diuretics, steroids, and postoperative course was uneventful without transplant rejection. He was also receiving oral administration of acetaminophen for lumbar disc hernia with no complaints. Severe pain with numbness from the right buttock to the toe developed suddenly, which was exacerbated by standing and walking. Intermittent claudication occurred approximately every 400 m. A diagnosis of lumbar disc hernia was made based on the positive results of the straight leg raising test and Kemp’s test beside the symptoms presented in the dermatome of the sciatic nerve by his previous physician.

We started trigger point block for lumbar erector spinae muscles and administration of oral pregabalin and tramadol. Repeated trigger point block and medication had little effect even at a dose of 300 mg/day for each.

**Fig. 1:**
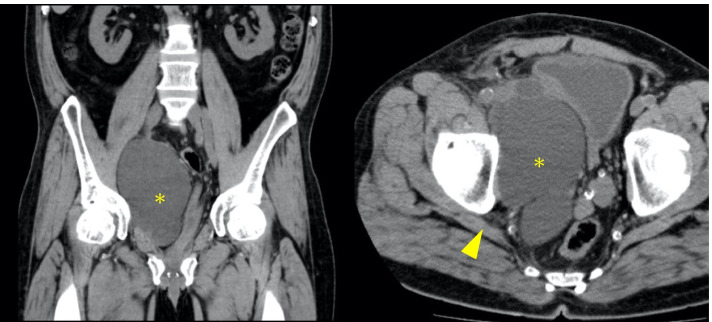
Computed tomography of the pelvis. The coronal scan shows a huge lymphocele with low signal intensity (*). The axial scan shows that a lymphocele (*) compresses the piriformis muscle (arrow)

One month after starting treatment, the symptoms exacerbated and the patient complained of continuous pain at rest and was unable to walk. The buttock pain was more intense, and a one-finger test for piriformis muscle syndrome was positive. Magnetic resonance imaging identified lumbar disc hernia of L4/5, which would have a small effect on sciatic neuralgia, suggesting the existence of extra-spinal disease. A urologist detected fluid accumulation around the transplanted kidney in the right abdomen on ultrasound examination and computed tomography revealed a large lymphocele (82 × 101 × 122 mm) in the transplanted renal stalk, compressing the sciatic nerve under the piriformis muscle (Fig. 1).

Percutaneous drainage and ethanol injection into the lymphocele were performed under fluoroscopic guidance by a radiologist. Forty milliliters of absolute ethanol in total divided into 20 mL, and injected in each 2 days. The lymphocele disappeared, and leg symptoms improved. Sciatica seemed to be caused by piriformis muscle syndrome of the lymphocele. Subsequently, the lymphocele and symptoms did not recur.

## Discussion

Lymphocele is a post-surgical complication for renal transplantation recipients, with an occurrence rate of 0.6-18.1% [5]. It is usually asymptomatic, and the incidence rate of symptomatic lymphocele is 5.2% [6]. It is often detected during imaging tests within 6 months of the transplant surgery, but is rarely discovered after several years. The risk factors for its incidence are injury of the lymph node around the iliac vein, immunosuppressants and steroids administration, and infection, among others [6]. Various symptoms, including abdominal pain, leg edema, and hydronephrosis, are caused by compression of the abdominal and pelvic organs. The typical leg symptom is unilateral edema caused by iliac vein compression [7, 8]. Sciatica without edema is an atypical symptom of a lymphocele after renal transplantation. Although there are some reports that lymphocele after radical prostatectomy compresses the external iliac vein and causes sciatica, there are no reports of piriformis muscle syndrome caused by a lymphocele after renal transplantation [7]. The treatment of pelvic lymphocele includes percutaneous drainage, sclerotherapy with alcohol, and laparoscopic surgery. The rate of recurrence after drainage and sclerotherapy is 31-37.5% [9]. Laparoscopic surgery is more efficient with fewer recurrences (4-8%). However, in our case, with drainage and sclerotherapy, the lymphocele was resolved completely and there was no recurrence.

Sciatica is a neuropathic pain commonly caused by lumbar spinal diseases, including spinal canal stenosis and lumbar disc hernia. Sciatica is caused by lumbar disc hernia in approximately 90% of the affected patients and rarely caused by extra spinal diseases such as malignant tumors, infections, and other neuropathic diseases [8]. Piriformis muscle syndrome is also a cause of sciatica, with an incidence of 6-36% [8]. Imaging tests are required for correct diagnosis of its etiology.

We misdiagnosed the cause of sciatica as a lumbar disc hernia because there was not a urological symptom at the first visit. The symptom of the pain appeared within 6 months after transplant surgery, and we should have imaging test at the earlier period.

Positive results of one finger test, suggestive of piriformis muscle one month after treatment initiation delayed the correct diagnosis.

In this case, the progressive symptoms met the criteria for the red flag signs of low back pain (severe continuous pain, use of immunosuppressive drugs, and steroids), indicating the existence of serious causes [9].

Lymphocele is a complication after renal transplantation and important for differential diagnosis of sciatica.

## Data Availability

Not applicable
